# Cardioprotective Potentials of Plant-Derived Small Molecules against Doxorubicin Associated Cardiotoxicity

**DOI:** 10.1155/2016/5724973

**Published:** 2016-05-23

**Authors:** Shreesh Ojha, Hasan Al Taee, Sameer Goyal, Umesh B. Mahajan, Chandrgouda R. Patil, D. S. Arya, Mohanraj Rajesh

**Affiliations:** ^1^Department of Pharmacology and Therapeutics, College of Medicine and Health Sciences, United Arab Emirates University, P.O. Box 17666, Al Ain, UAE; ^2^Department of Pharmacology, R. C. Patel Institute of Pharmaceutical Education and Research, Shirpur, Maharashtra 425405, India; ^3^Department of Pharmacology, All India Institute of Medical Sciences, New Delhi 110029, India

## Abstract

Doxorubicin (DOX) is a potent and widely used anthracycline antibiotic for the treatment of several malignancies. Unfortunately, the clinical utility of DOX is often restricted due to the elicitation of organ toxicity. Particularly, the increased risk for the development of dilated cardiomyopathy by DOX among the cancer survivors warrants major attention from the physicians as well as researchers to develop adjuvant agents to neutralize the noxious effects of DOX on the healthy myocardium. Despite these pitfalls, the use of traditional cytotoxic drugs continues to be the mainstay treatment for several types of cancer. Recently, phytochemicals have gained attention for their anticancer, chemopreventive, and cardioprotective activities. The ideal cardioprotective agents should not compromise the clinical efficacy of DOX and should be devoid of cumulative or irreversible toxicity on the naïve tissues. Furthermore, adjuvants possessing synergistic anticancer activity and quelling of chemoresistance would significantly enhance the clinical utility in combating DOX-induced cardiotoxicity. The present review renders an overview of cardioprotective effects of plant-derived small molecules and their purported mechanisms against DOX-induced cardiotoxicity. Phytochemicals serve as the reservoirs of pharmacophore which can be utilized as templates for developing safe and potential novel cardioprotective agents in combating DOX-induced cardiotoxicity.

## 1. Introduction

Doxorubicin (DOX) is a potent and widely used anthracycline antibiotic for the treatment of cancers. However, the major impeding issue pertaining to the clinical application of DOX is related to its ability to induce untoward toxicity to the healthy tissues [1]. The occurrence of fatal cardiotoxicity in pediatric as well as in adult patients is characterized by an irreversible cardiomyopathy which compromises the clinical utility of DOX and accounts for the major cause of the chemotherapy related morbidity and mortality [1]. In spite of introducing several less toxic derivatives of DOX, elicitation of cardiotoxicity still remains the major concern [2]. However, with the advent of newer class of monoclonal antibodies revolutionized cancer chemotherapy, still this approach is burdened with myriad adverse effects [3]. Thus, the use of traditional cytotoxic drugs continues to be a preferred mode for the treatment of cancer.

To limit the DOX-induced cardiotoxicity, several molecules, such as beta blockers, angiotensin receptor blockers, amifostine, dexrazoxane, Mesna (2-mercaptoethane sulfonate Na), leucovorin, and erythropoietin, have been evaluated as cardioprotective adjuvants in preclinical studies [4]. Recently, dexrazoxane, when subjected to clinical trial against combating DOX-induced cardiotoxicity, exhibited marked cardioprotection and did not compromise the anticancer activity of DOX [5]. Similarly, carvedilol (beta blocker) has also been demonstrated to confer protection against DOX-induced cardiotoxicity in human subjects [6]. However, large-scale clinical applications of these adjuvants are yet to be established in human subjects. The substantial burden arising from cancer and cardiotoxicity and their interrelationship in imposing morbidity and mortality has emerged as the major driving factor for the academia and pharmaceutical industry to devise and develop strategies that can simultaneously provide long-term cardioprotection from DOX-associated cardiotoxicity without compromising the efficacy of cancer chemotherapy [7]. Since the origin of the human civilization, plants and herbs have been traditionally used in the treatment of various diseases and ailments [8]. In this direction, the prospect of harnessing the potentials of plant-derived small molecules (phytochemicals) appears to provide rich dividends, since phytochemicals have been extensively studied in the preclinical studies and shown to possess anti-inflammatory, antioxidant, and anticancer activities. Phytochemicals are the natural constituents of herbs and plants. Moreover, anticancer drug paclitaxel and antimalarial agent artemisinin are phytochemicals originally extracted from plants; however, these agents are now chemically synthesized [9, 10].

**Figure 1:**
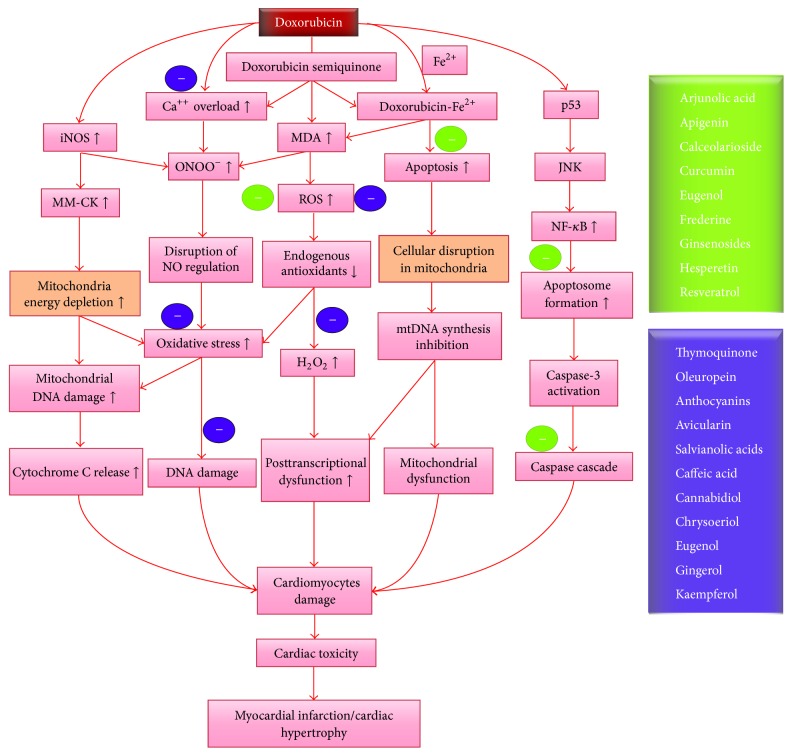
This scheme shows the pathways involved in the elicitation of DOX-induced adverse effects in the myocardium and its attenuation by phytochemicals.

In this review, we have systematically presented the evidence wherein the phytochemicals were investigated for cardioprotective effects against DOX and the relevant mechanisms. Several mechanisms have been postulated for the development of DOX-induced cardiotoxicity. However, oxidative stress driven inflammation, apoptosis, and myocardial remodeling have emerged to be the key players in DOX-induced cardiotoxicity. In-depth description of pathophysiology of DOX-induced cardiotoxicity has been reviewed elsewhere [11, 12]; however, to provide clarity to our discussion, we have provided a simplified scheme for the pathomechanisms in Figure 1.

## 2. Phytochemicals Limiting DOX-Induced Cardiotoxicity

In this section, we systematically described the phytochemicals investigated for their cardioprotective activity against DOX-induced toxic effects in the myocardium. Phytochemicals investigated in the animal model of DOX-induced cardiotoxicity are listed in Table 1 and the molecules investigated in cell culture models (*in vitro*) are presented in Table 2.

### 2.1. Arjunolic Acid

Arjunolic acid is a chiral triterpenoid saponin, isolated from* Terminalia arjuna.* Arjunolic acid treatment to adult rat cardiomyocytes in the presence of DOX attenuated caspase-dependent apoptotic signaling by ameliorating proapoptotic p53, p38, and JNK-MAPKs and mitochondrial pathways leading to apoptosis. Furthermore, arjunolic acid when administered to rats significantly inhibited DOX-induced myocardial toxicity by mitigating oxidative stress and apoptotic pathways [13].

### 2.2. Anthocyanins

Anthocyanins are a group of polyphenolic compounds which are abundantly found in common fruits and vegetables. The effect of six anthocyanidins (cyanidin chloride, delphinidin chloride, malvidin chloride, pelargonidin chloride, peonidin chloride, and petunidin chloride) and seven anthocyanins (cyanidin 3-O-*β*-galactopyranoside chloride, cyanidin 3-O-*β*-glucopyranoside chloride, delphinidin 3-O-*β*-glucopyranoside chloride, malvidin 3-O-*β*-glucopyranoside chloride, pelargonidin 3-O-*β*-glucopyranoside chloride, peonidin 3-O-*β*-glucopyranoside chloride, and petunidin 3-O-*β*-glucopyranoside chloride) was investigated against DOX-induced cardiotoxicity in H9c2 cardiomyoblasts. All these anthocyanidins improved the cell viability via quenching of reactive oxygen species (ROS) [14]. Delphinidin was found to confer protection against DOX and etoposide inhibition of topoisomerase II, thus warranting careful scrutiny against the use of these agents in combating DOX-induced cardiotoxicity.

### 2.3. Apigenin

Apigenin is a flavone type flavonoid predominantly found in flowers of chamomile. It is also present in edible plants such as fruits (oranges, apples, cherries, and grape fruits) and vegetables (onions, celery, parsley, broccoli, pepper, barley, and tomatoes) [15]. Apigenin treatment to adult rat cardiomyocytes significantly improved cell viability in the presence of DOX and the mechanism appeared to involve quenching of ROS, mitigation of lipid peroxidation, and myocyte necrosis [16]. However, to date, no studies have demonstrated apigenin efficacy* in vivo*.

### 2.4. Avicularin

Avicularin, chemically a biflavonoid and a quercetin glycoside, is isolated from the leaves of a flowering plant,* Malus hupehensis*, popularly known as Chinese crabapple, Hupeh crab, or tea crabapple [17]. The protective effects of avicularin against DOX-induced cardiotoxicity were demonstrated in H9c2 cells and the mechanism purported was via its antioxidant property [17]. However, the cardioprotective effects of avicularin are yet to be confirmed* in vivo*.

### 2.5. Berberine

Berberine is an alkaloid identified in the roots and bark of the* Berberis* species [18]. For the first time, its protective effects on DOX-induced cardiotoxicity in mice were studied by Lv et al. [19]. Berberine was found to reduce mortality, improve body weight and cardiac function, and restore ECG changes in DOX-treated rats. Furthermore, in neonatal rat cardiomyocytes, berberine inhibited DOX-induced apoptosis via counteracting ROS induced p53 activation, mitochondrial dissipation, executioner caspase activation, and activation of AMPK [19]. Moreover, berberine has also been shown to inhibit DOX-induced cardiotoxicity via mitigation of biotransformation of DOX, thereby limiting the bioavailability of doxorubicinol (a major alcohol metabolite of DOX) in the cytoplasm of rat hearts [20].

### 2.6. Baicalein

Baicalein is a flavonoid derived from the roots of* Scutellaria baicalensis* [21]. By using cultured chick cardiomyocytes* in vitro*, it was shown that baicalein attenuated DOX-induced ROS activation, proapoptotic MAPK, and apoptosis [22]. Recently, in a murine model of DOX-induced cardiotoxicity, baicalein significantly mitigated cardiac injury via augmenting nuclear factor E2-related factor-2 (Nrf2), antioxidant defense, blunting of nitrative stress, inflammation, and apoptosis [23].

### 2.7. Caffeic Acid Phenethyl Ester (CAPE)

Caffeic acid phenethyl ester is an active component of propolis which is the major hive product of bees and is known to be rich in flavonoids [24]. CAPE has been demonstrated to attenuate DOX-induced cardiotoxicity via attenuation of ROS generation and apoptosis. Furthermore, it also improved cardiac function as assessed by hemodynamic measurements and preserved the myocardial structure [25].

### 2.8. Calceolarioside

Calceolarioside is a phenylpropanoid glycoside isolated from* Calceolaria hypericina* known to possess antiplatelet and anticancer activities [26]. Calceolarioside attenuated DOX-induced cardiotoxicity in H9c2 cells via upregulation of antioxidant enzymes and suppression of apoptosis [27]. However, these effects are yet to be confirmed in animal models.

### 2.9. Cannabidiol

Cannabidiol (CBD) is a major nonpsychoactive constituent of the plant* Cannabis sativa*, popularly known as Marijuana and used for recreational as well as medicinal purposes [28]. In a chronic model of DOX-induced cardiotoxicity in rats, CBD has been demonstrated to suppress myocardial toxicity via attenuating oxidative stress, inflammation, and cell death pathways [29]. Recently, Hao et al. demonstrated that CBD attenuated DOX-induced cardiotoxicity via augmenting mitochondrial biogenesis and blunting of oxidative and nitrative stress and apoptosis [30]. It is also pertinent to note that CBD also exerts several cardioprotective actions against diabetic cardiovascular complications [31] and it has been approved in Canada and Europe for the management of pain associated with multiple sclerosis [32].

### 2.10. Carotenoids

Carotenoids are the organic pigments and constitute a large group of more than 600 compounds found in plants, which impart color to the leaves and fruits [33]. These are produced from 8 isoprene molecules and contain 40 carbon atoms and are also known as tetraterpenoids with polyisoprenoid structure having a long conjugated double bond system forming the backbone of the molecule, which may be terminated by cyclic end groups that contain oxygen-bearing substitutes. The electron-rich conjugated system of the polyene is believed to afford antioxidant and free radical scavenging activity and these pharmacological effects were attributed to health benefits offered by carotenoids [33]. Recently, the cardioprotective efficacy of carotenoids was demonstrated in DOX-induced tumor-bearing mice [34]. Specifically, carotenoids were found to improve the antioxidant defense and preserve the myocardial membranes, reflected as reduced leakage of myocyte injury marker enzymes without compromising DOX activity on the tumor growth inhibition [34].

### 2.11. Chrysin

Chrysin is a flavone class of flavonoid and one of the most important bioactive constituents of different fruits, vegetables, and mushrooms [35]. Recently, chrysin cardioprotective effect against DOX-induced acute cardiotoxicity in rats was demonstrated by Mantawy et al. [36]. Chrysin was found to improve antioxidant defense, attenuate oxidative/nitrative stress, and suppress the generation of inflammatory mediators [36].

### 2.12. Catechins

Epigallocatechin-3-gallate (EGCG) accounts for 50–80% of catechins in green tea and represents about 200–300 mg in a brewed cup of green tea. Convincing data are available to demonstrate that catechins possess potent antioxidant, anti-inflammatory, immunomodulatory, cardioprotective, and anticancer activities [37]. Green tea leaf extract supplementation in cultured rat cardiomyocytes showed its ability to protect the cells against DOX-induced decreased H9c2 cells viability, via quenching of ROS generation [38]. EGCG* in vitro* has been demonstrated to protect cardiomyocytes of neonatal rat hearts from DOX-induced cytotoxicity by attenuating ROS production, apoptosis, and increasing activities and protein expression of endogenous antioxidant enzymes [39]. In another study, EGCG treatment to rat cardiomyocytes significantly attenuated DOX-induced ROS generation and alterations in myocyte contractile dynamics via modulation of proteins involved in calcium handling system [40]. In addition, EGCG also elicited cardioprotective effects on a chronic model of DOX-induced cardiotoxicity via attenuation of oxidative stress and apoptosis pathways [41].

It has been reported that supplementation of EGCG in the diet increases the activities of P-450 family of reductase, augments the bioavailability of DOX, and could predispose the subject to increased risk of cardiotoxicity [42]. Hence, further studies are warranted to investigate and extend the cardioprotective benefits of ECGG, without untoward perpetuation of DOX-induced cardiotoxicity.

### 2.13. Chrysoeriol

Chrysoeriol is a flavone compound isolated from the leaves of* Digitalis purpurea*, popularly known as Foxglove and well reputed for its cardioprotective actions [43]. Chrysoeriol has been found to reduce cell death and attenuate ROS generated oxidative stress and lipid peroxidation in DOX-induced cardiotoxicity in H9c2 cardiomyoblasts without affecting antitumor activity of DOX [43]. For an evidence based approach, additional studies on its cardioprotective efficacies are warranted.

### 2.14. Curcumin

Curcumin is a phenolic yellow pigment constituent found in the rhizomatous parts of* Curcuma longa* (turmeric) [44]. Several* in vitro* and* in vivo* studies have demonstrated curcumin cardioprotective actions against DOX-induced myocardial toxicity. The key mechanisms postulated for curcumin cardioprotective activity include diminution of oxidative stress, inflammation, and associated cell death pathways [45–48]. Although curcumin has been reported to elicit several beneficial effects in various preclinical studies, the bioavailability is yet to be established in human subjects. Therefore, derivatives of curcumin are being perused to increase its bioavailability and, in this direction, a recent report suggests that the nanoparticle of curcumin could ameliorate DOX-induced cardiotoxicity [49].

### 2.15. Eugenol

Eugenol is the active component of essential oil isolated from* Syzygium aromaticum*, popularly known as clove, which is one of the common ingredients of spice mixtures [50]. Eugenol treatment was shown to significantly improve antioxidant defense mechanisms, decrease lipid peroxidation, and attenuate abnormal Ca^2+^ transients in the cardiomyocytes along with inhibition of apoptosis in rat hearts following acute DOX administration. Eugenol also preserved the myocardium and restored hemodynamics along with preserved histology [51].

### 2.16. Frederine

7-Monohydroxyethylrutoside (monoHER2, frederine) is a synthetic flavonoid, and it significantly inhibited DOX-induced myocardial toxicity, via suppression of oxidative stress and apoptosis in a chronic murine model [52, 53]. In addition, monoHER2 did not interfere with DOX anticancer activity* in vitro* and* in vivo* [54]. Considering these observations, monoHER2 could be further developed for this clinical application as cardioprotective adjuvant.

### 2.17. Gingerol

Gingerol is the pungent phenolic constituent of* Zingiber officinalis* (ginger) [55]. In a chronic model of DOX-induced cardiomyopathy, gingerol inhibited DOX-induced myocardial ROS generation, inflammation via attenuation of NF-*κ*B activation, and downregulation of soluble receptor for advanced glycation end products (sRAGE). In addition, gingerol also inhibited myocardial apoptosis via mitigating caspase-3 activities [56].

### 2.18. Ginsenosides

Ginsenosides are the saponin constituents of* Panax notoginseng*, popularly known as ginseng in traditional Chinese medicine [57]. Ginsenosides have been classified into protopanaxatriols (Rg1, Rh1, and PPT) and protopanaxadiols (Rg3, Rh2, and PPD). Of the ginsenosides, protopanaxadiols such as ginsenoside Rb1, ginsenoside Rh2, and compound K have been shown to exhibit anticancer and anti-inflammatory activities [57].

Ginsenoside Rh2 (Rh2) has been shown to elicit cardioprotective effects against DOX-induced cardiotoxicity in H9c2 cell line, as well as* in vivo* in an acute mouse and chronic rat model of DOX-induced cardiomyopathy. Rh2 enhanced cell viability of H9c2 cells and ameliorated DOX-induced release of the CK-MB, LDH. Furthermore, Rh2 ameliorated DOX-induced myocardial toxicity in mouse and rats via suppressing oxidative stress and improved the indices of cardiac function as determined by ECG [58].

### 2.19. Diosgenin

Diosgenin is a steroid saponin found abundantly in several plants including* Solanum* and* Dioscorea* species and* Costus speciosus*. In a chronic model of DOX-induced cardiomyopathy, diosgenin elicited cardioprotective effects, via activation of prosurvival kinase, protein kinase A (PKA), diminution of p38-MAPK, caspase-3 activities, and generation of free radicals along with attenuation of inflammatory mediators. Mechanistically, it was found to improve myocardial fibrosis and increase the cardiac levels of cGMP via modulation of phosphodiesterase-5 activity [59].

### 2.20. Hydroxybetulinic Acid

23-Hydroxybetulinic acid is isolated from* Pulsatilla chinensis.* In a chronic murine model of DOX-induced cardiomyopathy, 23-hydroxybetulinic acid significantly improved the survival of the animals and inhibited apoptosis mainly via inhibition of DOX metabolism in the mitochondria. Similar results were also obtained in H9c2 cells [60].

### 2.21. Hesperidin

Hesperidin is a bioflavonoid abundantly found in vegetables and citrus fruits such as oranges, lemons, and grapefruits [61]. Citrus flavonoids have been shown to reduce risk of cardiovascular diseases prominently due to their antioxidant and anti-inflammatory effects involving numerous cell signaling pathways [61]. It has been found to improve antioxidant status, inhibit lipid peroxidation, and reduce myocardial enzyme leakage by salvaging myocardium in DOX-induced cardiotoxicity in acute toxicity model conducted rats [62]. Additionally, it sensitized cancer cells to DOX-induced apoptosis and showed synergism in inhibiting P-gp and multidrug resistance, thus appearing to be effective as an adjunct to enhance the efficacy and attenuate the resistance to DOX during chemotherapy [63]. Thus, the potential of citrus flavonoids as cochemotherapeutic and cardioprotective agents is encouraging but further studies are warranted for conclusive evidence in cancer as well as chemotherapy associated cardiotoxicity. In addition, hesperetin (aglycone) derivative of hesperidin also ameliorated chronic DOX treatment associated cardiotoxicity in rats, via attenuation of p38-MAPK, caspase-3, and NF-*κ*B activation and oxidative stress in the myocardial tissues [64].

### 2.22. Hydroxytyrosol

Hydroxytyrosol is a polyphenolic constituent in* Olea europaea*, popularly known as olive oil, which is widely used in food and medicine [65]. Hydroxytyrosol has been shown to improve cardiac function by maintaining homeostasis at mitochondrial level, by preserving mitochondrial electron transport chain complexes I–IV and inhibiting apoptosis-inducing factor, and oxidative stress markers in chronic DOX-induced cardiotoxicity in rats harboring breast cancer [65]. Furthermore, hydroxytyrosol did not compromise the DOX antitumor activity against the implanted tumor cells and also improved the survival of the animals [65].

### 2.23. Isorhamnetin

Isorhamnetin is a flavonol aglycone abundantly found in several medicinal plants, such as* Hippophae rhamnoides* (sea buckthorn) [66]. Isorhamnetin significantly conferred cardioprotection in a chronic model of DOX-induced cardiotoxicity. The mechanism of cardioprotection involves suppression of oxidative stress and activation of mitochondrial apoptotic pathway and mitogen-activated protein kinase pathways, suggesting antioxidant mediated cardioprotective mechanism. Similar results were obtained in H9c2 cells [66]. Furthermore, it also synergistically improved the DOX anticancer activity in tumor cell lines [66].

### 2.24. Indole-3-carbinol

Indole-3-carbinol is a natural indole compound predominantly found in cruciferous vegetables [67]. In a chronic DOX-infusion associated cardiotoxicity murine model, indole-3-carbinol was found to reduce solid Ehrlich tumor size and volume, augment antioxidant defense, and inhibit lipid peroxidation, leading to stabilization of cell membrane and reduced leakage of myocyte injury marker enzymes. It was also found to decrease sphingosine kinase 1 (SphK1) activity and inflammatory mediators along with mitigating histological perturbations and modulating cell death mediators [67].

### 2.25. Kaempferol

Kaempferol is one of the most common dietary flavonoids and it is well studied for its antiapoptotic, cardioprotective, antioxidative, anti-inflammatory, chemopreventive, and anticancer properties as well as modulation of chemoresistance [68]. The cardioprotective effects of kaempferol against DOX-induced cardiotoxicity in rats were demonstrated using a chronic model. Kaempferol counteracted cardiotoxicity by inhibiting p53 expression in mitochondrion-dependent apoptotic signaling and ERK-dependent mitogen-activated protein kinase pathway following binding to the promoter region of the Bax proapoptotic gene. It also effectively suppressed DOX-induced extracellular signal-regulated kinase (ERK1/2) activation but had no effect on p38 and JNK [69].

### 2.26. Lycopene

Lycopene is a carotenoid and nonprovitamin A found abundantly in* Lycopersicum esculentum* (tomatoes) and known to impart color to tomatoes [33]. Karimi et al., in a very early study, demonstrated the protective effect of tomato extract and lycopene on acute DOX-induced cardiotoxicity in mice [70]. Tomato extract and lycopene prevented rise in myocyte injury marker enzyme, CK-MB, in serum and ameliorated cardiomyocytes injury evidenced by histopathological examination. Furthermore, Yilmaz et al. studied the protective role of lycopene in DOX-induced heart and kidney toxicities using biochemical and histopathological assessments and reported that lycopene has potential to inhibit lipid peroxidation and improve antioxidants evidenced by reduced lipid peroxides and improved GSH in both the heart and the kidneys. The protective effect was further substantiated by histopathological changes. The authors concluded that treatment with lycopene might prevent cardiac and renal toxicities in rats [71]. However, the results were not further substantiated by functional improvement as lycopene did not prevent left ventricular systolic dysfunction induced by DOX [72]. However, it suppressed DOX-induced myocyte damage without preventing interstitial collagen accumulation increase [72].

Lycopene supplementation also increased lycopene absorption in heart, liver, and plasma and, in another study, the same group of authors showed that there was no depletion of lycopene from myocardium of lycopene-supplemented rats treated with DOX and that higher antioxidant capacity in myocardium and less oxidative cleavage of lycopene in intestinal mucosa of DOX-treated rats suggest an antioxidant role of DOX rather than acting as a prooxidant [73]. The authors further showed that tomato-oleoresin enhances the chemotherapeutic effect of DOX. It maintained lycopene levels in heart and protected against cardiac oxidative DNA damage induced by DOX in rats [73]. The lycopene protected the heart against DOX associated cardiotoxicity by several mechanisms including the quenching of singlet oxygen, peroxy radicals, reaction with free radicals, restoring levels of vitamin E and vitamin C, reducing DNA damage, restoring cellular antioxidants, and preventing depletion of glutathione. Several studies showed the potential role of lycopene in the prevention of side effects of antineoplastic drugs in cell culture and animal models [74]. These results suggest that tomato extract and lycopene inhibit DOX cardiotoxicity and collectively might serve as a novel combination chemotherapeutic agent with DOX to limit free radical-mediated organ injury. However, further studies are required to investigate the role of lycopene in mitigating the side effects of chemotherapy in human subjects.

### 2.27. Luteolin-7-O-*β*-D-glucopyranoside

Luteolin-7-O-*β*-D-glucopyranoside is a flavonoid isolated from the plant* Dracocephalum tanguticum* that is widely used in Chinese and Tibetan traditional medicine [75]. The cytoprotective activities were demonstrated on DOX-induced cytotoxicity in H9c2 cardiomyocytes. Among several isolated compounds, luteolin-7-O-*β*-D-glucopyranoside was found to show antioxidant effect and a potent cytoprotective activity against DOX-induced toxicity as evidenced by decreased death of H9c2 cells, reduced myocyte injury marker enzymes, and reduced intracellular concentration of ROS and Ca^2+^ [75]. Recently, Yao et al. also demonstrated protective effects of luteolin-7-O-glucoside on DOX cytotoxicity in H9c2 cells [76]. It was found to improve cell viability and ameliorate ROS generation and mitochondrial depolarization. Furthermore, it enhanced the expression of prosurvival kinases and diminished ROS generation [76].

### 2.28. Morin Hydrate

Morin hydrate is a biflavonoid commonly found in fruits such as guava, fig, almond, grapes, and apple and vegetables such as onion, seed weeds, and several other members of Moraceae family [77]. It has been observed to enhance antioxidant defense against oxidative stress in human umbilical vein endothelial cells (ECV304) and HepG2 cells and minimize DOX toxicity in ECV304 and primary mouse cardiomyocytes [77]. However,* in vivo* studies regarding morin efficacy in combating DOX-induced cardiotoxicity are still lacking.

### 2.29. Mangiferin

Mangiferin is a xanthonoid structure with C-glucosyl linkage and polyhydroxy component found in many plant species; however,* Mangifera indica* (mango tree) is the major source [78]. Using a chronic model of cardiomyopathy in rats, mangiferin has been shown to exert cardioprotective action against DOX-induced cardiotoxicity by inhibition of proinflammatory mediators and proapoptotic genes and regulating calcium homeostasis modulating proteins [79].

### 2.30. Naringin

Naringin is a flavanone glycoside abundantly found in citrus fruits such as lemon, oranges, and grapefruits and in tomatoes. It has been documented to possess numerous biological properties such as antioxidant, anti-inflammatory, and antiapoptotic activities [80]. In an acute model of DOX-induced cardiotoxicity, naringin treatment improved antioxidant defense and inhibited lipid peroxidation along with histopathological preservation, thereby reducing leakage of myocyte marker enzymes. It also decreased the levels of inflammatory mediators and restored the mitochondrial complexes (I–IV) activities in the heart tissues along with histopathological salvage [80]. The studies indicate cardioprotective effects; however, further clinical research is required to provide significant insights into the mechanisms underlying the effects of naringin on human subjects.

### 2.31. Naringenin and Its Derivatives

Naringenin is a flavanone commonly found in citrus fruits such as grapefruit, orange, and lemon [80]. Arafa et al. demonstrated that naringenin elicited antioxidant mediated protection against DOX-induced cardiac toxicity in Swiss albino rats [81]. Recently, the combination of p-coumaric acid and naringenin was found to be superior in exerting antioxidant mediated cardioprotection against DOX-induced cardiotoxicity in rats [82]. Additionally, naringenin enhanced antitumor effect of DOX by selectively modulating drug efflux pathways; that is, it inhibited the activity of multidrug resistance-associated protein and did not affect the* in vivo* pharmacokinetics of intravenously administered DOX [80].

Naringenin-7-O-glucoside is a flavanone glycoside isolated from* Dracocephalum rupestre*. It has been demonstrated to protect against DOX-induced cardiotoxicity in H9c2 cells [83]. Particularly, naringenin-7-O-glucoside improved cell viability, prevented the release of myocyte injury marker enzymes LDH and CK, and augmented antioxidant defense [84]. Furthermore, naringenin-7-O-glucoside was observed to enhance NAD(P)H: quinone oxidoreductase (NQO1) and ERK activation and Nrf2 protein levels in DOX stressed H9c2 cells. These phenotypic changes brought about by naringenin-7-O-glucoside are attributed to the induction of antioxidant defense and attenuation of cell death pathways [85]. However,* in vivo* studies are yet to be performed.

### 2.32. Ocotillol

Ocotillol is an aglycone derivative of pseudoginsenoside-F11, which is devoid of sugar moiety and is found in American ginseng,* Panax quinquefolius*. Fu et al. reported that ocotillol enhances survival rate of animals in both acute and chronic models of DOX-induced cardiotoxicity. Ocotillol prevented depletion of glutathione and lipid peroxidation along with restoration of myocyte injury marker enzymes following preservation of cardiomyocytes cell membrane. Furthermore, ocotillol also improved cardiac function and hence was suggested as an adjuvant for counteracting DOX-induced cardiotoxicity [86].

### 2.33. Oleuropein

Oleuropein is a phenolic constituent of* Olea europaea* (olive oil) [87]. Andreadou et al. have shown the protective effect of oleuropein against DOX-induced acute cardiotoxicity in rats. Oleuropein was found to restore the myocardial necrosis marker enzyme levels and attenuation of oxidative stress and apoptosis [88]. Moreover, the same group of authors in a separate study demonstrated that oleuropein treatment aids the compensation of distressed energy metabolic pathways mechanistically by restoration of metabolites to the normal levels as DOX generated free radicals nonenzymatically convert pyruvate to acetate and alpha-ketoglutarate to succinate [87]. The cardioprotective role of oleuropein in chronic DOX-induced cardiomyopathy has also been demonstrated [89]. Particularly, oleuropein treatment significantly suppressed DOX-induced oxidative/nitrative stress, augmented prosurvival kinases, and improved the cardiac functions [89].

### 2.34. Osthole

Osthole is a coumarin compound found in several medicinal plants such as* Cnidium monnieri* and* Angelica pubescens* [90]. In rat neonatal cardiomyocytes, osthole significantly improved the survival of the cells by abrogating apoptosis, wherein the mechanism appeared to involve the suppression of mitochondrial pathway of apoptosis triggered by DOX [90]. However,* in vivo* studies have not been conducted, and it is warranted to recapitulate the* in vitro* cardioprotective actions of osthole.

### 2.35. p-coumaric Acid

p-coumaric acid is a phenolic acid that serves as a precursor of other phenolic compounds and is found in plants such as peanut, tea, and coffee [82]. p-coumaric acid has been shown to attenuate oxidative stress and inhibit myocyte injury in DOX-induced myocardial injury in rats [91]. In a separate study, p-coumaric acid in combination with naringenin showed cardioprotection by augmentation of antioxidant defense against DOX-induced cardiotoxicity in rats [82].

### 2.36. Periplogenin

Periplogenin is a cardenolide isolated from* Aegle marmelos*, commonly known as Bael in the traditional Indian system of medicine [92]. Periplogenin has been shown to decrease lipid peroxide levels, improve antioxidant defense, and salvage cardiomyocytes in a chronic model of DOX-induced cardiotoxicity in rats [92].

### 2.37. Plantainoside D

Plantainoside D is an iridoid glucoside isolated from* Picrorhiza scrophulariiflora* (Picrorhiza). The chemopreventive and antioxidant activities encouraged evaluating the cardioprotective effect of plantainoside D against DOX-induced apoptosis in H9c2 cells. Plantainoside D was found to inhibit oxidative stress and proinflammatory cytokines expression and attenuate apoptosis in H9c2 cardiomyoblasts [93].

### 2.38. Phycocyanin

C-Phycocyanin is a biliprotein found in* Spirulina platensis*, blue-green algae. Phycocyanin has been found to protect against DOX-induced oxidative stress and apoptosis in adult rat cardiomyocytes as evidenced by reduced ROS formation, DNA fragmentation, and attenuation of Bax as well as release of cytochrome C and increase in the activity of caspase-3 [94]. However, these* in vitro* findings are yet to be confirmed in rodent model of DOX-induced cardiomyopathy.

### 2.39. Proanthocyanidin and Derivatives

Proanthocyanidins are a mixture of structurally and functionally diverse chemicals which are predominantly found in grape seed and show high bioavailability and protect the organs from toxic chemicals used to induce diseases in* in vitro* and* in vivo* studies [95, 96]. Ray et al. reported the bioavailability and protective property of grape seed proanthocyanidin and a novel IH636 grape seed proanthocyanidin extract against DOX-induced cardiotoxicity as well as multiorgan protection in mice [97]. Bagchi et al. further reported that IH636 proanthocyanidin extract afforded protection was superior to vitamin C, vitamin E, and beta-carotene and demonstrated significant cytotoxicity towards human breast, lung, and gastric adenocarcinoma cells, while enhancing the growth and viability of normal cells in both* in vitro* and* in vivo* studies [98]. In another study, proanthocyanidins enhanced DOX-induced antitumor effect and reversed drug resistance and mechanisms attributed partially to the promotion of DOX-induced apoptosis through elevation of intracellular DOX, Ca^2+^, and Mg^2+^ concentration and reduction of pH value and mitochondrial membrane potential in DOX-resistant K562/DOX cells [99]. Furthermore, proanthocyanidin strongly enhanced the antitumor activity of DOX and ameliorated chronic DOX-induced myocardial oxidative stress and immunosuppression in tumor-bearing mice [99]. In addition, grape seed proanthocyanidin also showed antioxidant mediated cardioprotection against both high and low dose DOX-induced cumulative chronic cardiotoxicities in rats [100].

### 2.40. Resveratrol

Resveratrol, a natural phytoalexin, is commonly found in* Vitis vinifera* (grapes) [101]. Resveratrol induced antioxidants and phase 2 enzymes in the H9c2 cells, accompanied by increased resistance to oxidative and electrophilic cell injury [102]. Additionally, there was no significant effect of resveratrol on NADPH-cytochrome P-450 reductase (P-450 reductase), which plays an important role in the metabolism of many endogenous compounds and xenobiotics including DOX. The enzyme P-450 reductase activates them to their more toxic metabolites via one electron reduction which triggers free radical cascade. In some cases, however, such transformation is essential to produce therapeutic effect of anticancer drugs [42].

In DOX-induced cardiotoxicity in rats, resveratrol has been shown to ameliorate the severity of cardiac dysfunction and prevent oxidant stress responses [103, 104]. Furthermore, resveratrol was found to confer cardioprotection and reduce cardiac fibrosis in acute as well as chronic* in vivo* models of DOX-induced cardiomyopathy in rats. Mechanistically, resveratrol has been demonstrated to protect against DOX-induced oxidative stress through changes in mitochondrial function, specifically the Sirt1 pathway, leading to cardiac cell survival [105]. Resveratrol attenuated DOX-induced cardiomyocyte apoptosis in mice via upregulation of Sirt1-mediated p53 deacetylation and activation of Sirt1, a NAD^+^-dependent deacetylase, resulting in improved mitochondrial function, which culminates in activation of the transcription factors which coordinate expression of key antioxidant proteins by binding to the antioxidant response elements that regulate cell survival [106]. The overexpression of Sirt1 inhibited cell apoptosis by suppression of p38-MAPK phosphorylation and caspase-3 activation along with amelioration of ROS generation and prevented DOX-induced functional loss in DOX-induced cardiomyocyte injury [106]. DOX induces autophagy in cardiomyocytes which is a degradation system for eukaryotic cells to turn over organelles and long-lived proteins, thereby maintaining cellular homeostasis. Thus, aberrant autophagy activity impairs basal cardiac structure and function, making animals more sensitive to stress-induced heart failure. The ability of resveratrol to inhibit autophagy is mediated by inhibition of p70S6 kinase 1 (S6 K1) that is essential for resveratrol to suppress DOX-induced autophagy and cytotoxic effects [107].

DOX inhibits AMP-activated protein kinase (AMPK), resulting in Sirt1 dysfunction and p53 accumulation in mouse embryonic fibroblasts, and pharmacological activation of AMPK by resveratrol has been shown to alleviate the side effects of DOX in H9c2 cells [108]. Furthermore, resveratrol has been shown to confer cardioprotection in DOX-induced cardiomyocyte apoptosis in nude mice by induction of heme oxygenase-1 (HO-1) mediated mechanisms [109, 110]. Resveratrol was also reported to aid the differentiation of adipose-derived mesenchymal stem cells to cardiomyocytes and protected against noxious effects of DOX to the myocardium [111]. Furthermore, resveratrol supplement along with exercise training was found to be more effective in preventing DOX-induced LV remodeling associated with the reduction of DOX-induced oxidative stress [112]. In spite of these advances made in preclinical studies, resveratrol bioavailability is seldom established in human subjects and this warrants further approaches to extend its beneficial effects to mankind [113].

### 2.41. Robinin

Robinin is a flavonoid glycoside isolated from leaves of* Vigna unguiculata*, a dietary plant used in traditional cuisine in India [114]. Treatment with robinin was found to improve endogenous antioxidants, reduce ROS generation, and inhibit lipid peroxidation and proinflammatory mediators such as cyclooxygenase (COX2) and lipoxygenase (LOX15) along with restoring myocyte injury marker enzymes. The improvement in the level of transforming growth factor-*β*1 (TGF-*β*1), Smad2, murine double minute (Mdm2), Smad3, cyclin-dependent kinase inhibitor 2A, Smad4, and Smad7 in addition to favorable modulation of p53, Bcl-2, and Bax revealed the cardioprotective mechanism of robinin in combating DOX-induced cardiotoxicity [114].

### 2.42. Rosmarinic Acid

Rosmarinic acid is an ester of caffeic acid abundantly found in numerous plants, being most common in Boraginaceae and Lamiaceae families [115]. Rosmarinic acid showed remarkable cytoprotection against DOX toxicity in neonatal rat cardiomyocytes and DOX-induced lipid peroxidation of heart membranes, mitochondria, and microsomes and effects were found to be comparable to dexrazoxane [116]. Furthermore, it inhibited DOX-induced oxidative stress and apoptosis in H9c2 cardiomyoblasts by improving cell viability, inhibiting the production of ROS, and activation of prosurvival kinases [115]. However,* in vivo* cardioprotective actions against DOX-induced cardiotoxicity are hitherto unknown.

### 2.43. Salvianolic Acids

Salvianolic acids especially salvianolic acid A and salvianolic acid B are the most abundant water-soluble compounds extracted from* Salvia miltiorrhiza* (Danshen or red sage) [117]. Salvianolic acid A has been shown to protect against DOX-induced mitochondrial toxicity* in vitro* in rat cardiomyocytes due to its antioxidant action, without antagonizing effect on the antitumor activity of DOX [118]. The protective effects of salvianolic acid were reconfirmed* in vivo* in another study, against DOX cardiotoxicity in mice [117], via abrogation of oxidative stress and inflammation.

### 2.44. Schisandrin B

Schisandrin B, a dibenzocyclooctadiene lignin, is isolated from the fruit of* Schisandra chinensis.* It has been shown to salvage cardiomyocytes, via conferring antioxidant defense by restoring glutathione flux in an acute animal model of DOX-induced cardiotoxicity [119]. Furthermore, it also mitigated DOX-induced cardiotoxicity in rabbits [120]. Recently, cardioprotective effects of schisandrin B against DOX-induced cardiotoxicity were reconfirmed and the mechanism of protection was evidenced by amelioration of proinflammatory cytokines, lipid peroxidation, DNA damage, apoptosis, and MAPK activation in the myocardial tissues [121, 122].

### 2.45. Salidroside

Salidroside, a phenylethanoid glycoside, has been isolated from the roots of* Rhodiola rosea* (Roseroot). Wang et al. demonstrated that treatment of salidroside to either H9c2 cells or mice stressed with acute DOX administration conferred cardioprotective effects. The mechanism was defined to involve antioxidant and suppression of proapoptotic mediators [123]. The cardioprotective effects of salidroside were reconfirmed in a placebo controlled clinical trial wherein sixty patients with breast cancer receiving epirubicin were given salidroside (600 mg/day) or placebo starting 1 week before chemotherapy and assessed at baseline and 7 days after each new epirubicin dose of 100 mg/m^2^. Decline in strain rate peak was observed at an epirubicin dose of 200 mg/m^2^, with no significant differences between salidroside and placebo. At increasing cumulative doses of epirubicin, the strain rate normalized only with salidroside, showing a significant difference in comparison with placebo at epirubicin doses of 300 mg/m^2^. The authors concluded that salidroside may provide protection against chemotherapy-induced early left ventricular regional systolic dysfunction in patients with breast cancer. Based on preclinical and clinical data, salidroside needs to be investigated further in a larger population for advocating salidroside as an adjuvant to thwart the DOX-induced cardiotoxicity.

### 2.46. Sesamin

Sesamin is a major lignin obtained from seeds of* Sesamum indicum*. Sesamin was observed to increase the endogenous antioxidant enzymes and prevent mitochondrial damage via activation of Sirt1 in an acute model of DOX-induced cardiotoxicity [124].

### 2.47. Sesamol

Sesamol is a phenolic constituent of oil obtained from seeds of* Sesamum indicum* and is used commonly as an edible oil. The cardioprotective effects of sesamol were confirmed in an* in vivo* study, wherein sesamol mitigated cumulative DOX-induced cardiomyopathy in rats [125]. Sesamol improved antioxidant defense status, reduced myocyte injury marker enzymes released from cardiomyocytes, and inhibited lipid hydroperoxide. The salvage of tissues evidenced by biochemical and histopathological studies demonstrated cardioprotective effects of sesamol [125]. However, further mechanistic studies should be carried out investigating the effect on DOX efficacy and pharmacokinetic interaction.

### 2.48. Silibinin

Silibinin, a flavonolignan, is an active component of* Silybum marianum* (milk thistle), popularly known as silymarin and known to constitute 50–70% of the silymarin extract. The cardioprotective effect exerted by silymarin, silibinin, dehydrosilibinin, and silychristin was comparable to that of dexrazoxane, while silydianin exerted the best protective effect [126].

### 2.49. Sulforaphane

Sulforaphane is an organosulfur compound found in a significant amount in cruciferous vegetables, especially in broccoli (*Brassica oleracea*) [127]. The cardioprotective effects of sulforaphane were first demonstrated in H9c2 rat myoblasts as evidenced by reduced number of apoptotic cells along with decreased expression of proapoptotic proteins such as Bax, caspase-3, and cytochrome C [128]. It also reduced ROS generation and restored mitochondrial membrane potential [128]. Moreover, the cardioprotective effects of sulforaphane were found to be mediated by the activation of the Kelch-like ECH-associated protein 1 (Keap1)/NF-E2-related factor-2 (Nrf2)/antioxidant-responsive element (ARE) pathway, which in turn mediates the induction of HO-1 [129].

### 2.50. Tanshinone IIA and Derivatives

Tanshinones are the group of bioactive compounds isolated from* Salvia miltiorrhiza* (Danshen), a Chinese medicinal plant reputed for the management of cardiovascular diseases in particular, angina pectoris, atherosclerosis, myocardial infarction, and ischemic-reperfusion injury [130]. Sodium tanshinone IIA sulfonate, a water-soluble derivative of tanshinone IIA, was demonstrated to be beneficial in reducing DOX-induced cardiotoxicity in mice hearts and in cultured cardiomyocytes [131]. Treatment with sodium tanshinone IIA sulfonate prevented decrease in body weight and reduced myocardial lipid peroxidation in mice along with improved activities of endogenous antioxidant enzymes. In addition, the antioxidative mechanism was also supported by* in vitro* experiments showing that sodium tanshinone IIA sulphonate scavenged DOX semiquinone free radical in heart homogenate and inhibited DOX-induced mitochondrial lipid peroxidation and swelling [131].

Furthermore, another study demonstrated the beneficial effect of tanshinone IIA on decreasing DOX-induced apoptosis in neonatal rat cardiomyocytes and underlying molecular mechanisms [132]. Tanshinone IIA ameliorated apoptosis and ROS generation induced by DOX in a dose-dependent manner. It was further supported by the inhibition of DOX-mediated reduction of the ratio of Bcl-2/Bax [132]. Furthermore, a separate study also recapitulated that tanshinone IIA significantly inhibited DOX-induced toxic effects in H9c2 cells as well as in animal models of cardiotoxicity [133]. In this study, tanshinone IIA was shown to improve cell viability and ameliorate apoptosis of DOX-induced cytotoxicity in H9c2 cells. Furthermore, the cardioprotective effects of tanshinone IIA sodium sulfonate were confirmed by decreased ST interval and QRS interval in ECG; improved histological appearance of myocardium; increased myocardial tensile strength; and decreased fibrosis [133]. Recently, Hong et al. evaluated the protective effect of tanshinone IIA on DOX-induced cardiomyocyte apoptosis and explored its intracellular mechanisms using primary cultured neonatal rat cardiomyocytes. Tanshinone IIA was found to inhibit DOX-induced reactive oxygen species generation, reduce the expression of caspase-3 and cytochrome C, and increase BcL-x(L) expression, resulting in protecting cardiomyocytes from DOX-induced apoptosis. In addition, tanshinone IIA also enhanced Akt phosphorylation in cardiomyocytes and inhibited apoptosis [134].

### 2.51. Tetrahydroxystilbene Glucoside

Tetrahydroxystilbene glucoside is one of the active components extracted from* Polygonum multiflorum* (knot grass). For the first time, Zhang et al. demonstrated its protective effect on neonate rat cardiomyocytes and on an acute mouse model of DOX-induced cardiotoxicity [135]. In the mouse model, it was shown to inhibit lipid peroxidation accompanying improved glutathione, reduced animal mortality, preserved histopathological changes, and restored levels of myocyte injury marker enzymes. In the* in vitro* study, it prevented DOX-induced loss of mitochondrial membrane potential, caspase-3 activation, and upregulation of Bax protein expression along with upregulation of Bcl-2 and inhibited ROS generation. It was also observed to inhibit DOX-induced increases in intracellular Ca^2+^ and apoptosis of cardiomyocytes in a concentration-dependent manner [135].

### 2.52. Thymoquinone

Thymoquinone is the main active constituent of the volatile oil of* Nigella sativa* Linn., popularly known as black seed, used for culinary and medicinal purposes [136]. Thymoquinone suppressed DOX-induced cardiotoxicity in an acute murine model of cardiomyopathy, without compromising antitumor activity of DOX [137]. Furthermore, thymoquinone also circumvented DOX-mediated cardiotoxicity in acute model, wherein the key mechanism was postulated to involve antioxidant pathways [138]. Finally, thymoquinone synergistically increased DOX activity in several cancer cell lines and prevented DOX-induced toxicity in H9c2 cells [139].

### 2.53. Tetrandrine

Tetrandrine is a bisbenzylisoquinoline alkaloid isolated from the dried root of* Stephania tetrandra*. In a chronic model of DOX-induced cardiomyopathy, tetrandrine significantly inhibited myocardial apoptosis via quenching of ROS and restoration of mitochondrial capacity. These beneficial effects were corroborated with improved indices of cardiac function [140]. It is pertinent to note that tetrandrine had a negligible effect in DOX pharmacokinetics properties in rodents, suggesting that tetrandrine might be a suitable candidate to be developed as cardioprotective adjuvant [141].

### 2.54. Z-Guggulsterone

Guggulsterone is a major active component of* Commiphora mukul,* popularly known as Guggul and reputed for its antihyperlipidemic and cardioprotective effects in Ayurvedic medicine [142]. Wang et al. demonstrated the protective activity of guggulsterone against DOX-induced cytotoxicity in H9c2 cells. It was found to improve cell viability, morphology, and cytotoxicity and cellular antioxidants along with inhibition of apoptosis by altering activity of PARP, caspase-3, cytochrome C release, and Bcl-2 proteins and reducing the activation of NF-*κ*B [143].

### 2.55. Vincristine

Vincristine is an alkaloidal constituent isolated from* Catharanthus roseus* (Madagascar periwinkle), also known as* Vinca rosea*. Recently, its potential to prevent DOX-induced cardiomyocyte death and related mechanisms has been reported in adult mouse cardiac myocytes [144]. Vincristine treatment to cardiomyocytes in the presence of DOX increased the cell viability. This was concordant with decreased PARP and caspase-3 activities and increased activation of prosurvival kinase Akt and diminished MAPK pathways [144]. However, the precise cardioprotective effects* in vivo* are yet to be demonstrated.

### 2.56. Visnagin

Visnagin is an active constituent isolated from fruit extracts of* Ammi visnaga* known as toothpick weed and used in traditional Chinese medicine for cardiovascular diseases [145]. Visnagin was recently shown to be cardioprotective in a zebrafish model of DOX-induced cardiomyopathy that recapitulates the cardiomyocyte apoptosis and contractility similar to those observed in humans [146]. Visnagin was found to rescue the cardiac performance and circulatory defects caused by DOX in zebrafish. It also attenuated DOX-induced apoptosis in cultured cardiomyocytes and* in vivo* in zebrafish and mouse hearts along with improved cardiac contractility in DOX-treated mice. Additionally, it did not interfere with DOX efficacy in several cultured tumor lines or in zebrafish and mouse xenograft models. Visnagin was observed to bind mitochondrial malate dehydrogenase (MDH2), a key enzyme in the tricarboxylic acid cycle that contributed to cardioprotection [146].

## 3. Concluding Remarks and Future Perspectives

From the analysis of the literature, it is evident that several phytochemicals exhibited cardioprotective effects* in vitro* and* in vivo* against DOX-induced cardiotoxicity. The key pathways modulated by phytochemicals in cardiomyocytes include oxidative stress, inflammation, and cell death pathways, as demonstrated in Figure 1. Majority of the phytochemicals were demonstrated to elicit cardioprotective activity in preclinical studies. However, they have not been translated for clinical utility in human subjects. The major impediment to the development of phytochemical based cardioprotective adjuvants pertains to their negligible pharmacokinetic actions in human subjects. Particularly, the poor or lack of bioavailability in human subjects retards the enthusiasm for further pharmaceutical development [113, 147].

In order to improve the bioavailability of phytochemicals, various synthetic derivatives have been pursued [44]. Although significant strides have been taken in delineating the pathomechanisms for DOX-induced cardiotoxicity, still we do not have bona fide clinical biomarker to predict early changes in the myocardium of patients who received DOX treatment [148]. Therefore, it is of paramount significance to devise a biomarker to predict the DOX-induced cardiotoxicity, because most patients (cancer survivors) exhibit dilated cardiomyopathy several years after exposure to DOX [148]. Furthermore, from Table 1, it is evident that there is discrepancy regarding the employment of appropriate models in studying phytochemicals protective effects in nullifying DOX-induced cardiotoxicity. Therefore, future studies addressing the phytochemicals protective effects against DOX cardiotoxicity should utilize the physiologically relevant cumulative (chronic) dosage regimen in rodents. In addition, future studies should obligatorily investigate the noninterference of phytochemicals against DOX anticancer activities in orthotropic tumor-bearing mouse models.

In sum, to exploit the true potentials of plant-derived compounds for drug development, significant intellectual and financial contributions are warranted from academia and pharmaceutical industry. Unfortunately, the major impediment in this direction is the lack of proper intellectual property rights protection that could protect the financial viability of the drug development projects based on phytochemicals for the treatment of cardiomyopathy. This caveat coupled with other pharmacodynamics and pharmacokinetic lapses pertaining to the phytochemicals precludes the attention of major pharmaceutical companies in their portfolio investments toward the drug development. However, academic research should be directed to develop phytochemicals derived small molecules with significant bioavailability in human subjects. Perhaps this approach could be envisaged for translational application in combating DOX-induced cardiotoxicity.

## Figures and Tables

**Table 1:** Phytochemicals investigated for cardioprotective activity against DOX-induced cardiotoxicity in *in vivo* studies.

Phytochemical	DOX-induced cardiomyopathy-animal model (acute or chronic)	Cardiac function determined (yes/no)	References
Arjunolic acid	Chronic	Yes	[13]

Berberine	Chronic	Yes	[20]

Berberine	Acute	Yes	[19]

Baicalein	Chronic	No	[23]

Caffeic acid phenethyl ester	Acute	Yes	[25]

Cannabidiol	Acute	Yes	[30]

Cannabidiol	Chronic	No	[29]

Carotenoids	Acute	No	[34]

Eugenol	Acute	Yes	[51]

Gingerol	Chronic	No	[56]

23-Hydroxybetulinic acid	Chronic	Yes	[60]

Hesperetin	Chronic	No	[64]

Hesperidin	Acute	No	[62]

Isorhamnetin	Chronic	No	[66]

Indole-3-carbinol	Chronic	No	[67]

Kaempferol	Chronic	No	[69]

Lycopene	Acute	No	[70]

Lycopene	Acute	No	[71]

Lycopene	Acute	Yes	[72]

Mangiferin	Acute	No	[79]

Mangiferin	Acute	No	[78]

Naringenin	Acute	No	[79]

Naringenin	Acute	No	[80]

Ocotillol	Acute	No	[86]

Ocotillol	Chronic	No	[86]

Hydroxytyrosol	Chronic	No	[65]

Tetrandrine	Chronic	Yes	[140]

Periplogenin	Chronic	No	[92]

p-coumaric acid	Acute	No	[82]

Procyanidins	Chronic	Yes	[100]

Robinin	Acute	No	[114]

Thymoquinone	Acute	No	[137]

Thymoquinone	Acute	No	[138]

Silibinin	Chronic	Yes	[126]

Sesamin	Acute	No	[124]

Sesamol	Chronic	No	[125]

Tetrahydroxystilbene glucoside	Acute	No	[135]

Oleuropein	Acute	No	[87]

Oleuropein	Acute	No	[88]

Oleuropein	Chronic	Yes	[89]

Frederine	Acute	Yes	[52]

Visnagin	Acute	Yes	[146]

Visnagin	Chronic	Yes	[146]

Schisandrin B	Acute	Yes	[121]

Schisandrin B	Chronic	Yes	[122]

Salvianolic acid A	Acute	Yes	[117]

Tanshinone IIA	Chronic	Yes	[133]

Oleuropein	Acute	No	[87]

Oleuropein	Acute	No	[88]

Oleuropein	Chronic	Yes	[89]

Frederine	Acute	Yes	[52]

Visnagin	Acute	Yes	[146]

Visnagin	Chronic	Yes	[146]

Schisandrin B	Acute	Yes	[121]

Schisandrin B	Chronic	Yes	[122]

Salvianolic acid A	Acute	Yes	[117]

Tanshinone IIA	Chronic	Yes	[133]

**Table 2:** Phytochemicals exhibiting cytoprotection in the *in vitro* models of DOX-induced cardiotoxicity.

Phytochemical	Concentration of the phytochemical	Cell culture model	DOX dose and time of incubation	References

Arjunolic acid	100 *μ*g/mL	Neonatal rat cardiomyocytes	1 *μ*M for 12 h	[13]

Apigenin	25–100 *µ*M	Rat heart cardiomyocytes	100 *µ*M for 8 h	[16]

Avicularin	10–80 *μ*M	H9c2 cells	20 *μ*M for 24 h	[17]

Berberine	0.06, 0.25, 1.0, and 4.0 *µ*M	Neonatal rat cardiomyocytes and MCF-7 cells	1 *µ*M for 2 h	[19]

Baicalein	25 *μ*M	Chick embryo cardiomyocytes and MCF-7 cells	1, 10, 50, or 100 *μ*M for 24 h	[22]

Calceolarioside	40 *μ*M	H9c2 cells	1, 2, or 5 *μ*M for 30 h	[27]

23-Hydroxybetulinic acid	0.2, 2, and 20 *μ*M	H9c2 cells	5 *μ*M for 18 h	[60]

Isorhamnetin	3.125 to 25 *µ*g/mL	MCF-7, HepG2, and Hep2 cells	1 *µ*M for 36 h	[66]

Kaempferol	5 to 50 *μ*M	H9c2 cells	1 *μ*M for 24 h	[69]

Morin hydrate	0.17 mM	ECV304 and HepG2 cells	6 mM for 12 h	[77]

Naringenin-7-O-glucoside	10–80 *μ*M	H9c2 cells	10 *μ*M for 24 h	[83]

Osthole	10–40 *μ*M	Neonatal rat cardiomyocytes	1 *μ*mol for 24 h	[90]

Luteolin-7-*O*-*β*-D-glucopyranoside	5, 10, and 20 *μ*M	H9c2 cells	20 *μ*M for 24 h	[75]

Luteolin-7-*O*-*β*-D-glucopyranoside	5–80 *µ*M	H9c2 cells	10 *µ*M for 24 h	[76]

Vincristine	10–30 *µ*M	Adult mouse cardiomyocytes	15 and 20 *µ*g/mL for 24 h	[144]

Sulforaphane	2.5 *µ*M	H9c2 cells	5 *µ*g/mL for 16–18 h	[129]

C-Phycocyanin	10 *μ*M	Adult rat cardiomyocytes	10 *μ*M for 4, 24, and 48 h	[94]

Plantainoside D	1–20 *μ*g/mL	H9c2 cells	1, 2, and 4 *μ*M for 30 h	[93]

Sesamol	12.5–50 *μ*M	H9c2 cells	1 *μ*M for 30 min	[125]

Tetrahydroxystilbene glucoside	3–300 *μ*M	Neonatal rat cardiomyocytes	1 *μ*mol/L for 24 h	[143]

Chrysoeriol	20 *µ*g/mL	H9c2 cells	1 *μ*M for 24 h	[43]

Visnagin	20 *μ*M	Neonatal rat and zebrafish cardiomyocytes, cardiac HL-1 cells	100 *μ*M for 48 h	[146]

Z-Guggulsterone	1–30 *μ*M	H9C2 cells	1 *μ*M for 24 h	[143]

Tanshinone IIA	0.1, 0.3, 1, and 3 *μ*M	Neonatal rat cardiomyocytes	1 *μ*M for 24 h	[134]

Tanshinone IIA	1.6–40 *μ*M	H9c2 cells	1 *μ*M for 24 h	[133]

Tanshinone IIA	0.5, 1, and 2 *μ*mol/L	Neonatal rat cardiomyocytes	1 *μ*mol/L for 24 h	[132]

Sodium tanshinone IIA sulphonate	0.05–0.5 mM	Mice heart mitochondria	0.2 mmol for 10 min	[131]

Anthocyanidins and anthocyanins	0–100 *μ*M	H9c2 cells and MCF-7 cells	1 *μ*M for 24 h	[14]

Caffeic, chlorogenic, and rosmarinic acid	100 and 200 *µ*M	Rat heart microsomes and mitochondria	100 *μ*M for 8 h	[116]

ECV304 cells: human umbilical vein endothelial cells; HepG2 cells: human hepatocellular carcinoma cells; MCF-7 cells: human breast carcinoma; H9c2 cells: rat ventricular cardiomyoblast cells.
